# Impaired reversal learning in the Dlg2+/− rat model of genetic risk for psychiatric disorder: Important questions regarding the neuro‐behavioral mechanisms of reversal learning

**DOI:** 10.1111/gbb.12870

**Published:** 2023-12-04

**Authors:** Tobias Bast, Rachel Grasmeder Allen, Silvia Maggi, Jacco Renstrom

**Affiliations:** ^1^ School of Psychology and Neuroscience@Nottingham University of Nottingham Nottingham UK

**Keywords:** Dlg2+/‐, reversal, rat, psychiatric disorders, hippocampus, schizophrenia, behavior, bowl digging, touch screen, animal model

## Abstract

In this issue, Griesius et al report that heterozygous Dlg2+/‐ rats showed a reversal learning impairment on a specific bowl‐digging task, whereas other reversal tasks were unaffected. The study suggests that Dlg2 gene disruption, which has been linked to neuropsychiatric disorders, including schizophrenia, may cause relatively specific impairments in reversal learning, an important aspect of cognitive flexibility. The study draws attention to two important issues regarding the neuro‐behavioral mechanisms of reversal learning, namely that hippocampal dysfunction, which is prominent in Dlg2+/‐ rats, may contribute to reversal learning impairments and that, depending on the task and previous experience, brain and behavioral mechanisms of reversal learning may differ.

Griesius et al^1^ report that heterozygous Dlg2+/− rats showed a specific reversal learning impairment on a deterministic substrate‐specific bowl‐digging task, whereas reversal on other bowl‐digging tasks (probabilistic substrate‐specific and both deterministic and probabilistic spatial) and a touch‐screen visual‐discrimination task was unaffected (alongside normal behavior on a range of other tests). The Dlg2 gene encodes the synaptic protein PSD3, and Dlg2 disruption has been associated with psychiatric disorders, including schizophrenia. The study suggests that Dlg2 gene disruption may cause relatively specific impairments in reversal learning, which is an important aspect of cognitive flexibility and disrupted in neuropsychiatric disorders, including schizophrenia.^2^, ^3^, ^4^ The findings raise two important questions.

First, which brain substrates underlie the reversal learning impairment? A main focus of neuroscience research on reversal learning has been on fronto‐striatal mechanisms, especially involving orbitofrontal cortex.^2^, ^4^ However, Griesius et al^1^ highlight that Dlg2+/− rats show reduced hippocampal synaptic integration and plasticity^5^ and propose this may contribute to reversal learning impairments (although Dlg2+/− rats probably have other neuronal dysfunction, outside the hippocampus, that may explain the impairments). Indeed, human^6^, ^7^ and animal model^8^, ^9^ studies have implicated the hippocampus in reversal learning (for a discussion of how hippocampal contributions to reversal learning may relate to theories of hippocampal function, see Reference 8). Overall, Griesius et al^1^ draw attention to the possibility that reversal learning may depend on brain substrates outside fronto‐striatal circuits, including hippocampal mechanisms, although this requires confirmation by future studies.

Second, why is one reversal task impaired, but not others? A strength of the study by Griesius et al^1^ is the use of different reversal tasks, with different procedural demands. Interestingly, Dlg2+/− rats only showed reversal impairments on one of these tasks. The task features (e.g., task difficulty/required training, sensory modality and type of discriminanda) explaining these different outcomes are difficult to pinpoint. It is possible that the specific reversal impairment on the substrate‐specific bowl‐digging task reflects that this task was tested first. Based on their first ‘reversal experience’, rats may form an expectation that reward contingencies of discriminanda can be reversed,^2^, ^9^ which may affect their behavioural strategies. Overall, on different tasks and depending on previous experience, rats may use different behavioural strategies to learn to reverse, and these strategies may depend on different brain substrates. Importantly, we have a limited understanding of which specific behavioural strategies, on a trial‐by‐trial basis, lead to successful reversal learning, which is typically indexed by measures taken across trials (e.g., trials to criterion). Recently developed Bayesian analytical approaches, which allow tracking of behavioural strategies with trial resolution,^9^, ^10^, ^11^ offer the opportunity to address this issue in future studies.

In summary, Griesius et al^1^ show that disruption of the Dlg2 gene can cause a relatively specific reversal learning impairment. Whether hippocampal synaptic dysfunction, which is prominent in the Dlg2+/− rats, contributes to reversal learning impairment remains to be determined. The study highlights that, depending on the task or previous experience, reversal learning may depend on different behavioural strategies and underlying brain substrates.
